# Supplementary material for: An open-source framework for large-scale, flexible evaluation of biomedical text mining systems
Source: J Biomed Discov Collab. 2008 Jan 29;3:1. doi: 10.1186/1747-5333-3-1 (PMC2276192; doi:10.1186/1747-5333-3-1)
Supplement: Additional file 1 — Evaluation platform source code. The additional file contains the software evaluation framework discussed in this paper. The most current release can be downloaded from SourceForge.net [1]. See the accompanying README file for instructions on installation and use. Also included in the distribution is a collection of UIMA wrappers for some commonly used BioNLP tools and annotated corpora. [file 1747-5333-3-1-S1.zip › bionlp_uima_repository-1.0.1/docs/api/src-html/edu/uchsc/ccp/uima/cc/summary/opendmap/TestPrinter.html]

```
001    /**
002     * The OpenDMAP for Protege Project
003     * February 2006
004     */
005    package edu.uchsc.ccp.uima.cc.summary.opendmap;
006    
007    import java.io.IOException;
008    import java.io.PrintStream;
009    import java.text.SimpleDateFormat;
010    import java.util.Date;
011    import java.util.Iterator;
012    
013    import org.apache.uima.cas.CAS;
014    import org.apache.uima.cas.CASException;
015    import org.apache.uima.collection.CasConsumer_ImplBase;
016    import org.apache.uima.collection.base_cpm.CasObjectProcessor;
017    import org.apache.uima.jcas.JCas;
018    import org.apache.uima.resource.ResourceInitializationException;
019    import org.apache.uima.resource.ResourceProcessException;
020    
021    import edu.uchsc.ccp.uima.annotation.CCPDocumentInformation;
022    import edu.uchsc.ccp.uima.util.opendmap.TestSummarizer;
023    
024    /**
025     * This class looks through the JCas for annotations of a certain type
026     * and prints them in a serialized format appropriate for later comparison
027     * using the TestChecker.
028     * <p>
029     * This code is used for automated testing.  The annotations
030     * are printed in a compact serialized fashion.
031     * <p>
032     * The parameters used are:
033     * <ul>
034     * <li> OutputToFile: Set to tru if the output should be written to a file.
035     *      By default output is written to standard output.
036     * <li> OutputFileName: The name for the output file if one is used.
037     * <li> OutputMentionTypes: A string of the form type,slot,slot,...;type,slot,slot, ... describing
038     *      the mention types and slots to include in the serialized output string.
039     * <li> FormatForWiki: Set to true if the output should be generated in
040     *      a format that enables it to be cut and pasted in the UCHSC wiki.
041     * </ul>
042     * 
043     * @author R. James Firby
044     */
045    public class TestPrinter extends CasConsumer_ImplBase implements CasObjectProcessor {
046            
047            /* The parameter names exposed to the UIMA descriptor for this CAS processor */
048            private static final String PARAM_OUTPUTTOFILE = "OutputToFile";
049            private static final String PARAM_OUTPUTFILENAME = "OutputFileName";
050            private static final String PARAM_OUTPUTMENTIONTYPE = "OutputMentionTypes";
051            private static final String PARAM_USEWIKIFORMAT = "FormatForWiki";
052            
053            /* The default for the 'Output To File' parameter */
054            private boolean outputToFile = false;
055            /* The default output stream */
056            private PrintStream mentionOutputStream = System.out;
057            /* The default value for the 'Format for Wiki' parameter */
058            private boolean useWikiFormat = false;
059            
060            /* The summarizer to create serialized output strings */
061            private TestSummarizer summarizer = null;
062    
063            /**
064             * The standard UIMA initialize method for this processor.  Get the parameter
065             * values and initialize the summarizer.
066             */
067      public void initialize() throws ResourceInitializationException {
068    
069            // Find out which SemanticAnnotation mentions we want to include
070            String mentionList = (String) getConfigParameterValue(PARAM_OUTPUTMENTIONTYPE);
071        summarizer = new TestSummarizer(mentionList);
072        
073            // See if we want to be writing to a file
074            outputToFile = false;
075            Boolean param = (Boolean) getConfigParameterValue(PARAM_OUTPUTTOFILE);
076            if (param != null)
077                    outputToFile = param.booleanValue();
078            
079        // If we are writing to a file, open it
080            mentionOutputStream = System.out;
081        if (outputToFile) {
082            String outputFilename = (String) getConfigParameterValue(PARAM_OUTPUTFILENAME);
083            if (outputFilename != null) {
084                  try {
085                    mentionOutputStream = new PrintStream(outputFilename);
086                  } catch (IOException ioe) {
087                      System.err.println("Error creating test printer output stream.");
088                      ioe.printStackTrace();
089                  }
090            }
091              }
092        
093        // See if we want to write the output in wiki format
094            useWikiFormat = false;
095            param = (Boolean) getConfigParameterValue(PARAM_USEWIKIFORMAT);
096            if (param != null)
097                    useWikiFormat = param.booleanValue();
098        
099        
100        // Write some header information
101        mentionOutputStream.println("## OpenDMAP UIMA Semantic Annotation Test Printer");
102        mentionOutputStream.println("## " + (new SimpleDateFormat()).format(new Date()));
103        if (mentionList != null)
104            mentionOutputStream.println("#! Mentions: " + mentionList);
105            
106              super.initialize();
107      }
108      
109      /**
110       * The standard UIMA process method.  Find the desired mentions in the JCas and
111       * print them out as serialized strings.
112       */
113      public void processCas(CAS cas) throws ResourceProcessException {
114        JCas jcas;
115        try {
116            jcas = cas.getJCas();
117        } catch (CASException e) {
118            System.err.println("Exception getting CAS in TestPrinter");
119            e.printStackTrace();
120            throw new ResourceProcessException(e);
121        }
122    
123        // Extract information about the Generif
124        Iterator docInfoIter = jcas.getJFSIndexRepository().getAnnotationIndex(CCPDocumentInformation.type).iterator();
125        String docID = "-1";
126        if (docInfoIter.hasNext()) {
127            CCPDocumentInformation docInfo = (CCPDocumentInformation) docInfoIter.next();
128            docID = docInfo.getDocumentID();
129        }
130        String docText = jcas.getDocumentText();
131        mentionOutputStream.println();
132        if (useWikiFormat) {
133          printWikiGenerif(mentionOutputStream, docID, docText);
134        } else {
135                mentionOutputStream.println("#!" + docID + ": " + docText);
136        }
137              mentionOutputStream.flush();
138                
139        // Print out all output annotations for this generif
140        for (String mentionString: summarizer.summarizeJCas(docID, jcas)) {
141            if (useWikiFormat) mentionOutputStream.print(" ");
142            mentionOutputStream.println(mentionString);
143        }
144            }
145      
146      /**
147       * Print a wiki format header for a generif.
148       * 
149       * @param stream The output stream.
150       * @param docId The document ID in the JCas (the Generif ID).
151       * @param docText The document text in the JCas (the Generif text).
152       */
153      private void printWikiGenerif(PrintStream stream, String docId, String docText) {
154            stream.print(" <font color=\"green\">");
155            stream.print(docId);
156            stream.print(": ");
157            int count = 1;
158            for (int i=0; i<docText.length(); i++) {
159                    char ch = docText.charAt(i);
160                    if ((ch == ' ') && (count > 70)) {
161                            stream.println();
162                            count = 0;
163                    } else {
164                            if (count == 0) for (int j=0; j<docId.length()+3; j++) stream.print(" ");
165                            stream.print(ch);
166                            count = count + 1;
167                    }
168            }
169            stream.println("</font>");
170      }
171    
172    }
173
```
